# S-nitrosylation of paired-related homeobox 1 promotes cardiac remodeling following myocardial infarction

**DOI:** 10.1016/j.redox.2025.103887

**Published:** 2025-10-30

**Authors:** Dashuai Wang, Shaoxuan Zhou, Yajing Tang, Zhenxing Liang, Xinyi Yu, Gongcheng Huang, Chen Huang, Shuangxi Wang, Hai Liu

**Affiliations:** a Department of Cardiovascular Surgery, The First Affiliated Hospital of Zhengzhou University, China; b Department of Interventional Radiology, The First Affiliated Hospital of Zhengzhou University, China; c State Key Laboratory for Innovation and Transformation of Luobing Theory, Key Laboratory of Cardiovascular Remodeling and Function Research, Chinese Ministry of Education, Chinese National Health Commission and Chinese Academy of Medical Sciences, Department of Cardiology, Qilu Hospital of Shandong University, Jinan, China

**Keywords:** Paired-related homeobox 1, S-nitrosylation, Fibroblast-to-myofibroblast differentiation, Cardiac remodeling, Myocardial infarction

## Abstract

**Backgrounds:**

Cardiac remodeling, mediated by fibroblast-to-myofibroblast differentiation, is a key pathophysiologic step to determine the prognosis of patients following myocardial infarction (MI). Paired-related homeobox 1 (Prrx1) is a master transcription factor of fibroblasts for myofibroblastic lineage progression. Protein S-nitrosylation by nitric oxide (NO) is highly related to regulate cellular functions. This study is to investigate whether and how Prrx1 S-nitrosylation plays a key role in postischemic remodeling of heart.

**Methods:**

The MI surgery was performed by ligation of left anterior descending coronary artery. Cardiac fibrosis was assessed using Masson staining. Heart function was measured by echocardiography.

**Results:**

MI induced cardiac remodeling as cardiac fibrosis and heart dysfunction in mice, accompanied with increased Prrx1 transcriptional activity, but inhibited by N-acetyl-cysteine administration. In recombinant human protein, NO donors increased Prrx1 S-nitrosylation at cysteine 207 (C207). In human cardiac fibroblasts, oxygen-glucose deprivation or transforming growth factor beta upregulated NO productions, Prrx1 S-nitrosylation, Prrx1 transcriptional activity, Wnt5a gene expression, and fibroblast-to-myofibroblast differentiation, which were abolished by Prrx1-C207R mutant. *In vivo*, exogenous expression of Prrx1-C209R alleviated MI-induced cardiac fibrosis and promoted the recovery of heart functions in mice. Fibroblast-specific Prrx1 gene knockout prevented cardiac fibrosis and heart dysfunctions in mice fowling MI. In human patients with post-MI, Prrx1 S-nitrosylation was increased.

**Conclusion:**

Upregulation of Prrx1 by S-nitrosylation increases Wnt5a gene expression to induce fibroblast-to-myofibroblast differentiation, which contributes to cardiac remodeling after MI. In perspective, targeting Prrx1 S-nitrosylation should be considered to improve the outcome of patients with MI.

## 1 Introduction

Myocardial infarction (MI) is a leading cause of sudden death worldwide [1,2]. In response to MI, myocardial fibrosis is caused by aberrantly activated cardiac fibroblasts, excessive accumulation of collagen fibers in the extracellular matrix of the heart muscle, increased collagen concentration, or imbalanced collagen composition [[3], [4], [5]]. Maintenance of proper ratio of myocardial interstitial collagen is important for the integrity of cardiac function [6,7]. Molecular mechanisms that underlie cardiac fibrotic disorders are still mostly unclear, and no specific therapies exist for treatment of myocardial fibrosis.

Under normal conditions, cardiac fibroblasts are scattered within the myocardium, maintaining the basic extracellular matrix. However, certain stimuli trigger cardiac fibroblasts to transdifferentiate into highly proliferative and migratory myofibroblasts, which express excessive smooth muscle α-actin (α-SMA) and have contractile capacities [8]. This process is called fibroblast-to-myofibroblast differentiation (FMD). Recently, a master transcription factor constructing core-regulatory circuitry, paired-related homeobox 1 (Prrx1), which determines the fibroblast lineage with a myofibroblastic phenotype, is identified for the fibroblast subgroup [9]. In vascular system, Prrx1 is involved matrix modulation [10] and inhibits adipogenesis through the upregulated expression of transforming growth factor beta (TGF-β) ligands [11,12]. However, the regulation of Prrx1 in ischemia-induced cardiac remodeling is currently unknown.

Protein S-nitrosylation conveys a large part of the ubiquitous influence of nitric oxide (NO) on cellular signal transduction, and accumulating evidence indicates important roles for S-nitrosylation both in normal physiology and in a broad spectrum of human diseases [[13], [14], [15]]. Generally, S-nitrosylation is a reversible, covalent addition of NO moiety to cysteine thiol, forming S-nitrosoprotein, which participates in the pathogenesis of health and diseases [16]. S-nitrosylation participates in the pathogenesis of various cardiovascular diseases, including cardiac hypertrophy and atherosclerosis [[17], [18], [19], [20]].

Therefore, we hypothesized that activation of Prrx1 by S-nitrosylation promotes FMD to induce cardiac fibrosis following MI. Here, we reported that ischemia triggers Prrx1 S-nitrosylation to upregulate the transcriptional activity, resulting in the gene expression of Wnt5a, a key regulator to determine cell differentiations of cardiac fibroblasts [21]. Mechanistically, oxygen-glucose deprivation (OGD) or TGF-β induces FMD through Prrx1 S-nitrosylation and subsequent Wnt5a expression in cardiac fibroblasts. In perspective, inhibition of Prrx1 S-nitrosylation is an effective approach to improve cardiac remodeling in patients with ischemic heart diseases.

## 2 Materials and Methods

An expanded Materials and Methods section is available in Online Supplements.

### 2.1 Animals and establishment of MI model

Mice at 8–12 weeks of age with 20–25 g body weight were used in this study. The surgery of MI was operated by ligation of left anterior descending coronary artery (LADCA) as described previously [22,23]. Cardiac remodeling including myocardial fibrosis and heart functions was detected at the 28th postoperative day. Welfare-related assessments, measurements and interventions were carried out before, during and after the experiment. This study was carried out in strict accordance with the recommendations in the Guide for the Care and Use of Laboratory Animals of NIH. The animal protocol was reviewed and approved by the Animal Care and Use Committee, Zhengzhou University.

During the chronic period following MI surgery, all animals were housed in a controlled environment with adequate amounts of food and water provided in individually ventilated cages with same size and wood shavings as bedding material. All animals were kept in a specific pathogen-free facility. At the end of the study, animals that underwent MI surgery were humanely killed by CO_2_ euthanasia and the condition of death was confirmed by cervical dislocation.

### 2.2 Echocardiography

Echocardiography with standard parasternal and apical views was conducted in the left lateral recumbent position as described previously [24]. Left ventricular ejection fraction (LVEF) and fractional shortening (LVFS) were calculated.

### 2.3 Statistical analysis

All quantitative results are expressed as mean ± SD. Multiple comparisons were analyzed with a one-way ANOVA followed by Tukey's HSD test or Dunnett's test. Comparisons between two groups were analyzed by unpaired Student's *t*-test between two groups. *P* < 0.05 was considered significant.

## 3 Results

### 3.1 Ischemia induces cardiac remodeling and protein S-nitrosylation in mice

**Fig. 1:**
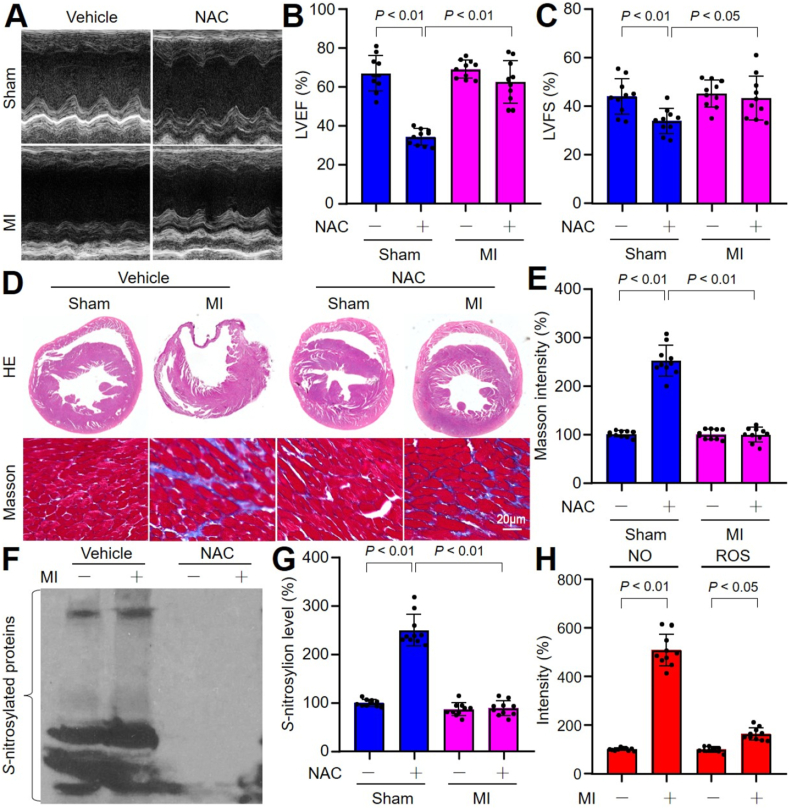
**Myocardial infarction (MI) induces cardiac remodeling through S-nitrosylation in mice.** The experimental protocol was illustrated in Online Fig. 1A. (**A-C**) The heart functions were determined at the 28th postoperative before sacrificed using ultrasound in **A**. LVEF in **B** and LVFS in **C** were calculated. (**D** and **E**) Hearts were harvested from mice to perform HE staining and Masson staining in **D**. Collagen content was calculated in **E**. (**F** and **G**) Protein S-nitrosylation was measured heart tissues using the biotin-switch method in **F** and quantification in **G**. (**H**) The productions of NO and ROS were determined in hearts. N = 10 per group. A one-way ANOVA followed by Tukey's HSD test was used to determine *P* value between two groups in **B, C, E**, and **G.** An unpaired Student's *t*-test was used to determine *P* value in **H**.

To test the hypothesis, as shown in Online Fig. 1A, we generated the MI model in mice by performing the surgery of LADCA ligation to induce post-ischemic cardiac remodeling [[25], [26], [27]]. There were obvious heart dysfunctions, as decreased LVEF and LVFS, at the 28th postoperative day in mice following MI surgery, compared to sham mice (Fig. 1A–C). The accumulations of collagen fibers, measured by Masson staining, were significantly higher in the hearts of mice with MI surgery than sham surgery (Fig. 1D and E). These data suggest that ischemia induces cardiac remodeling in mice.

### 3.2 Ischemia induces FMD and protein S-nitrosylation in mice

FMD is responsible to cardiac fibroblasts by expressing α-SMA and producing excessive collagen I (Col I) and collagen III (Col III), which account for more than 90 % of the total amount of myocardial interstitial collagen following a variety of myocardial injuries [8]. [28,29]. Thus, we the detected the protein levels of α-SMA, Col I and Col III in the heart. As indicated in Online Fig. 1B–E, the levels of α-SMA, Col I and Col III, such as mRNA and protein in post-ischemic hearts were increased in hearts after ischemia, consistent with other previous reports [30]. Importantly, there were increased protein S-nitrosylation (Fig. 1F and G) in ischemic area in the hearts.

### 3.3 N-acetyl-cysteine (NAC) administration improves cardiac remodeling and FMD in mice following MI

NO is a highly reactive gas molecule that may cause protein dysfunction through S-nitrosylation [31]. To determine whether S-nitrosylation contributes to MI-induced cardiac remodeling, we pretreated mice with NAC, a precursor of glutathione synthesis to prevent S-nitrosylation [32], for 2 weeks prior to 4-week ischemia (Online Fig. 1A). As expected, in mice with post-MI, NAC dramatically prevented cardiac remodeling as improved heart functions (Fig. 1A–C) and limited cardiac fibrosis (Fig. 1D and E), accompanied with the inhibitions of FMD (Online Fig. 1B–E) and S-nitrosylation (Fig. 1F and G).

SH group within NAC potentially reacts with NO, which is required for both 3-nitrotyrosine and *S*-nitrosylation [33]. Thus, we measured the levels of NO and reactive oxygen species (ROS) in the hearts from mice with sham or MI surgery. As indicated in Fig. 1H, ROS production was lightly increased in mice with MI (approximately 1.5 times), while NO production was dramatically increased (approximately 5.0 times). Taking these data together, it reveals that MI promotes FMD-mediated cardiac remodeling mainly through induction of protein S-nitrosylation.

### 3.4 NO donor induces Prrx1 S-nitrosylation *in vitro*

**Fig. 2:**
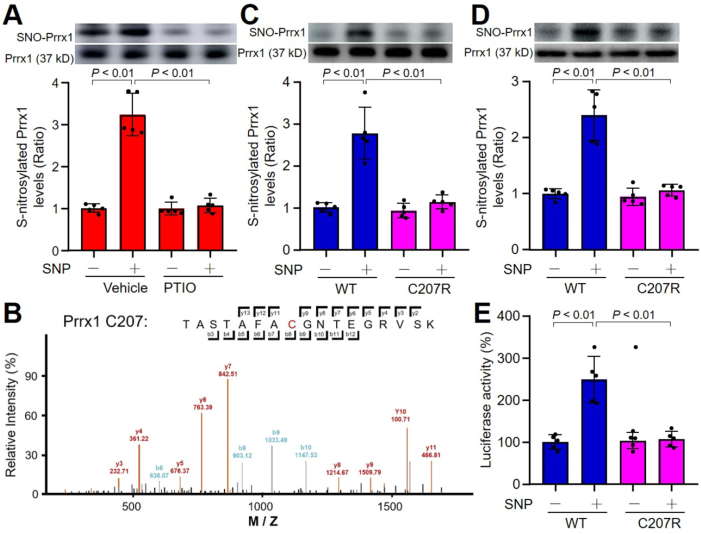
**Nitric oxide (NO) S-nitrosylates human Prrx1 protein at C207 and activate Prrx1 transcriptional activity.** (**A**) Recombinant human Prrx1 protein was incubated with four NO donors (1 mM) for 2 h in reaction buffers. Reaction products were subjected to determine Prrx1 S-nitrosylation. *P < 0.05 *vs.* Vehicle. (**B**) Illustration of human Prrx1 C207 S-nitrosylation identified by mass spectrometry. (**C**) Purified human Prrx1 (*WT*, C207R) proteins from *E.coli*. were incubated with sodium nitroprusside (SNP) for 2 h in reaction buffers. Reaction products were subjected to determine Prrx1 S-nitrosylation. (**D** and **E**) Cardiac fibroblasts were transfected with plasmids of Prrx1 (*WT*, C207R) for 48 h and then incubated with SNP (1 mM) for 2 h. Total cell lysates were subjected to detect Prrx1 S-nitrosylation in **D** and transcriptional activity using luciferase reporter gene in **E**. N = 5 per group. A one-way ANOVA followed by Dunnett's test was used to determine *P* value in **A**. A one-way ANOVA followed by Tukey's HSD test was used to determine *P* value between two groups in **C**, **D** and **E**.

Prrx1 is a master transcription factor of stromal fibroblasts for myofibroblastic lineage progression [9]. We thought that Prrx1 contributes to cardiac fibrosis through NO-mediated protein S-nitrosylation. Thus, we performed the analysis of amino acid sequence of Prrx1 protein to identify whether Prrx1 protein is S-nitrosylated. As shown in Online Fig. 2A, there are two cysteines (C207 and C208) in human Prrx1 protein and one cysteine (C209) in mouse Prrx1 protein, suggesting that Prrx1 is potentially S-nitrosylated. To this end, purified recombinant human Prrx1 protein was incubated with NO donor SNP or plus NO scavenger carboxy-PTIO (Online Fig. 2B). As indicated in Fig. 2A, *in vitro* exposure of recombinant Prrx1 protein to SNP increased Prrx1 S-nitrosylation. Further, NO scavenger PTIO blocked the effects of SNP, demonstrating that Prrx1 protein can be post-translationally modified through NO-directed S-nitrosylation.

### 3.5 Prrx1 protein is S-nitrosylated at cysteine 207 in human or 209 in mouse

As reported [34,35], Prrx1 occurs as two isoforms, that is, pmx-1a and pmx-1b, that are produced by alternative splicing. The first 199 amino acid residues have identical sequences, but the C-terminal regions differ between the two iso-forms. The longer isoform of 245 amino acid residues, pmx-1b (also known as Prrx1A, encoded by pmx-1b, NM_022716), has an OAR (otp, aristaless, and rax) domain in its C-terminal region (amino acid residues 200–245), whereas the shorter isoform of 217amino acid residues, pmx-1a (also known as Prrx1B, encoded by pmx-1a, NM_006902), lacks this domain but contains a repressor domain (amino acid residues 200–217).

To identify the S-nitrosylation site of Prrx1, we performed mass spectrometry analysis (Online Fig. 3A) and detected the 207th amino acids (C207) in human Prrx1, which is equal to C209 in mouse Prrx1, was S-nitrosylated (Fig. 2B). The C207 locates in OAR domain at the C-terminal region and is highly conserved across different species (Online Fig. 3B and C). To confirm Prrx1 C207 S-nitrosylation, we generated plasmids expressing His-Prrx1-WT or His-Prrx1-C207R with replacements of cysteine to arginine (C to R) without affecting the basic function, and transfected these DNA constructs into *E.coli*. for expression. Purified Prrx1 proteins were indicated with SNP *in vitro*. As depicted in Fig. 2C, SNP increased S-nitrosylated level of His-Prrx1-WT protein, but not His-Prrx1-C207R protein, demonstrating that NO induces Prrx1 S-nitrosylation at C207.

**Fig. 3:**
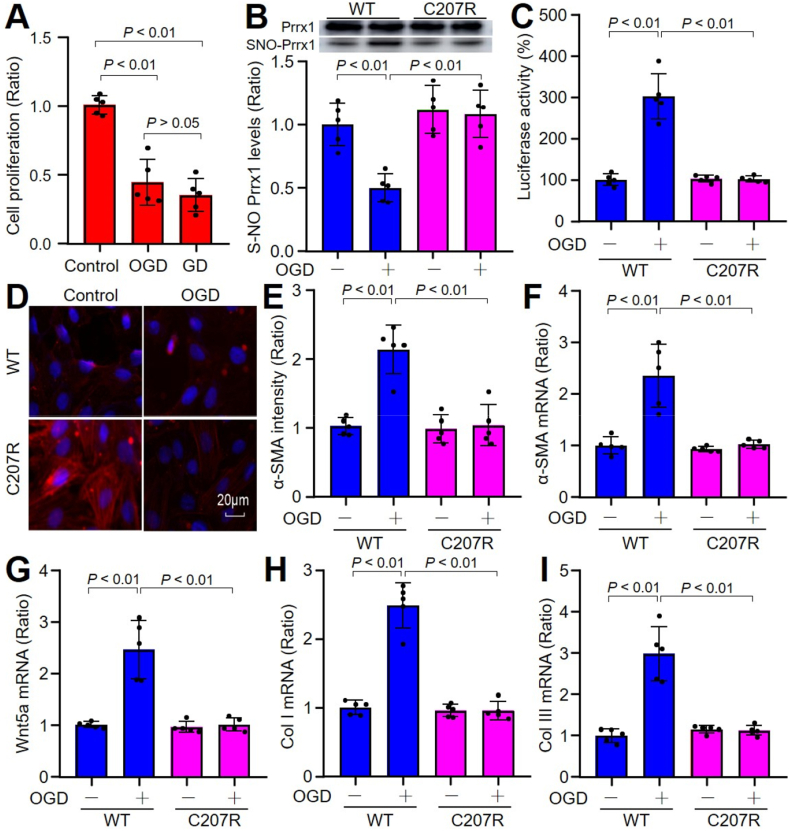
**Oxygen-glucose deprivation (OGD) induces Prrx1 S-nitrosylation to promote fibroblast-to-myofibroblast differentiation in cardiac fibroblasts.** Cardiac fibroblasts were transfected with plasmids of Prrx1 (*WT*, C207R) for 48 h followed by OGD or glucose deprivation (GD) for 24 h. (**A**) Cell proliferation was measured using CCK8. (**B** and **C**) Total cell lysates were subjected to detect Prrx1 S-nitrosylation in **B** and transcriptional activity using luciferase reporter gene in **C**. (**D** and **E**) IFC analysis of α-SMA in **D** and quantification in **E** were performed. (**F–I**) Gene expressions of α-SMA in **F**, Wnt5a in **G**, Col I in **H**, and Col III in **I** were determined using quantitative PCR. N = 5 per group. A one-way ANOVA followed by Tukey's HSD test was used to determine *P* value between two groups.

### 3.6 S-nitrosylation activates Prrx1 transcriptional activity in fibroblasts

Prrx1 is a transcriptional factor to upregulate target-gene expressions through a putative Prrx1 binding site (ACAATTTC) such as Wnt5a, which is a key regulator to determine cell differentiations of cardiac fibroblasts [21]. We generated and transfected fibroblasts with plasmids of His-Prrx1-WT and His-Prrx1-C207R followed by SNP treatment. As shown in Fig. 2D and E, in cells expressing His-Prrx1-WT, SNP increased S-nitrosylated level of His-Prrx1-WT protein and Prrx1 transcriptional activity measured by luciferase reporter gene analysis, but not His-Prrx1-C207R protein, demonstrating that NO activates through Prrx1 S-nitrosylation at C207.

### 3.7 Oxygen-glucose deprivation (OGD) via Prrx1 S-nitrosylation induces FMD in cardiac fibroblasts

To identify the role of Prrx1 S-nitrosylation in the regulation of FMD, we transfected cardiac fibroblasts with plasmids of His-Prrx1-WT and His-Prrx1-C207R followed by OGD, which mimics ischemia *in vivo*. As provided, OGD, which is similar to glucose deprivation on cell proliferation (Fig. 3A), increased Prrx1 S-nitrosylation and transcriptional activity (Fig. 3B and C), upregulated the levels of α-SMA mRNA and protein (Fig. 3D–F), and enhanced gene expression of Wnt5a, Col I, and Col III (Fig. 3G–I) in cells expressing His-Prrx1-WT, rather than cells with enforced expression of His-Prrx1-C207R, showing that OGD-induced FMD is Prrx1 S-nitrosylation dependent.

### 3.8 TGF-β induces FMD in cardiac fibroblasts through Prrx1 S-nitrosylation

**Fig. 4:**
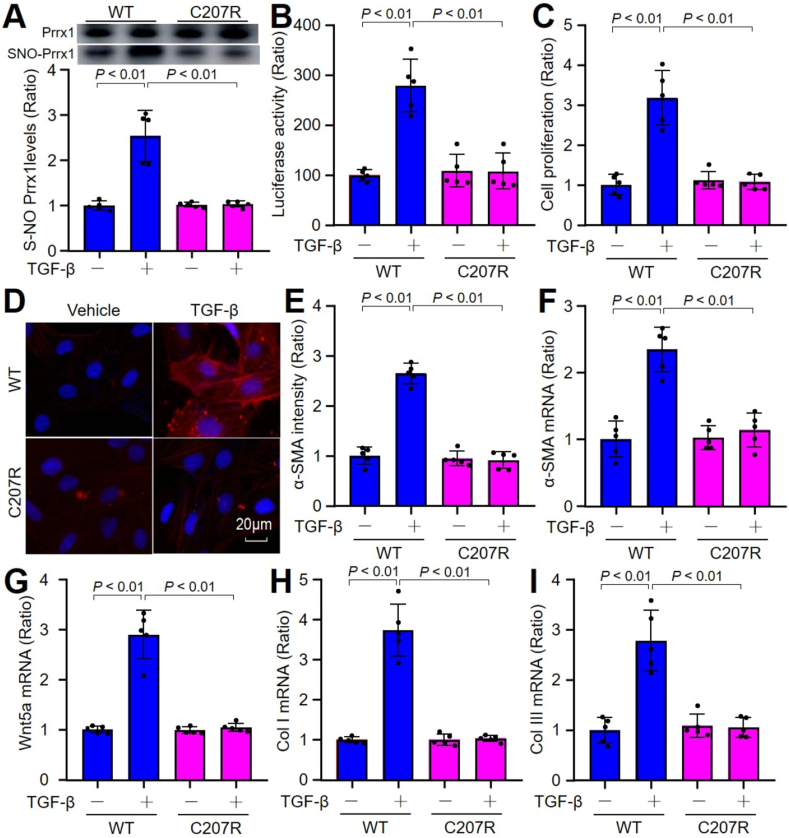
**Transforming growth factor beta (TGF-β) promotes fibroblast-to-myofibroblast differentiation of cardiac fibroblasts through Prrx1 S-nitrosylation.** Cardiac fibroblasts were transfected with plasmids of Prrx1 (*WT*, C207R) for 48 h and then incubated with TGF-β (10 ng/ml) for 24 h. (**A** and **B**) Total cell lysates were subjected to detect Prrx1 S-nitrosylation in **A** and transcriptional activity using luciferase reporter gene in **B**. (**C**) Cell proliferation was measured using CCK8 in **C**. (**D** and **E**) IFC analysis of α-SMA in **D** and quantification in **E** were performed. (**F–I**) Gene expressions of α-SMA in **F**, Wnt5a in **G**, Col I in **H**, and Col III in **I** were determined using quantitative PCR. N = 5 per group. A one-way ANOVA followed by Tukey's HSD test was used to determine *P* value between two groups.

Previous studies have demonstrated the predominant involvement of TGF-β in many cardiac fibrotic conditions [21,36]. Therefore, we determined the role of Prrx1 S-nitrosylation in the regulation of FMD induced by TGF-β. Similar to OGD, TGF-β increased Prrx1 S-nitrosylation and activity (Fig. 4A and B), promoted cell proliferations (Fig. 4C), both mRNA and protein levels of α-SMA (Fig. 4D–F), and gene expressions of Wnt5a, Col I, and Col III (Fig. 4G–I) in cells expressing His-Prrx1-WT, but not in cells with enforced expression of His-Prrx1-C207R. Collectively, it suggests that Prrx1 S-nitrosylation mediates FMD of cardiac fibroblasts induced by TGF-β.

### 3.9 Wnt5a gene knockdown abolishes Prrx1-mediated FMD in fibroblasts

**Fig. 5:**
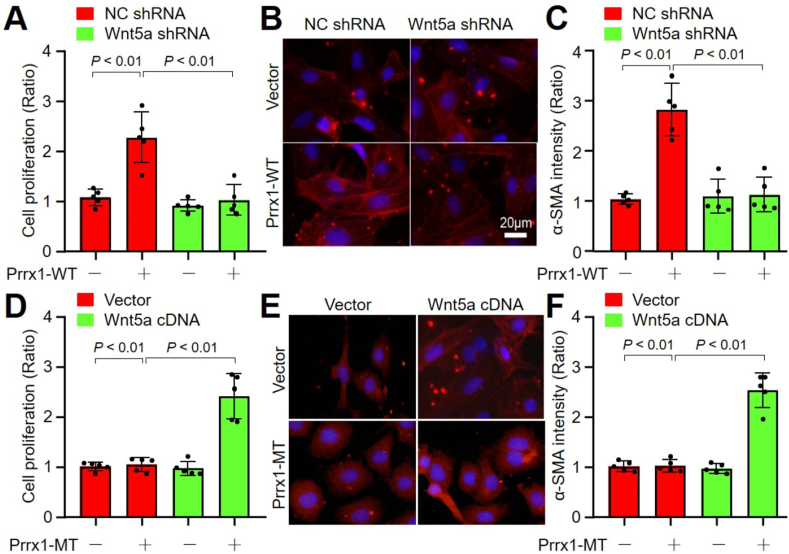
**TGF-β-induced Prrx1-mediated fibroblast-to-myofibroblast differentiation is Wnt5a dependent in cardiac fibroblasts**. (**A-C**) Cardiac fibroblasts were infected with adenovirus expressing Prrx1-WT plus negative control (NC) or Wnt5a shRNA for 48 h followed by TGF-β (10 ng/ml) treatment for 24 h. Cell proliferation was measured using CCK8 in **A**. IFC analysis of α-SMA in **B** and quantification in **C** were performed. (**D-F**) Cardiac fibroblasts were infected with adenovirus expressing Prrx1-MT (C207R) plus vector or Wnt5a cDNA for 48 h followed by TGF-β treatment for 24 h. Cell proliferation was measured using CCK8 in **D**. IFC analysis of α-SMA in **E** and quantification in **F** were performed. N = 5 per group. A one-way ANOVA followed by Tukey's HSD test was used to determine *P* value between two groups.

Since Wnt5a is a target gene of paired-related homeobox family to determine cell differentiations of cardiac fibroblasts [21], and knowing that FMD is Prrx1 S-nitrosylation dependent, we next elucidated whether the Prrx1-mediated FMD is Wnt5a dependent. To this end, cardiac fibroblasts were infected with adenovirus expressing His-Prrx1-WT plus Wnt5a shRNA followed by TGF-β treatment. As expected, though TGF-β increased cell proliferation and α-SMA protein level (Fig. 5A–C) and gene expressions of α-SMA, Col I and Col III (Online Fig. 4A) in His-Prrx1-WT cells expressing negative control shRNA, but not in cells expressing Wnt5a shRNA.

### 3.10 Wnt5a overexpression promotes Prrx1-mediated FMD in fibroblasts

Contrariwise, we infected cardiac fibroblasts with adenovirus expressing His-Prrx1-MT (C207R) plus Wnt5a CDNA followed by TGF-β treatment. The effects of TGF-β on cell proliferation and α-SMA protein level (Fig. 5D–F), and gene expressions of α-SMA, Col I and Col III (Online Fig. 4B) were not observed in cells expressing Prrx1-MT, but rescued by Wnt5a overexpression. In one word, these data reveal that the FMD of cardiac fibroblast is regulated by the Prrx1/Wnt5a signaling.

### 3.11 TGF-β affects NO-related signaling in cardiac fibroblasts

We also performed the omics analysis to observe the effects of TGF-β in cardiac fibroblasts. Differentially expressed genes were analyzed by RNA sequencing (Supplementary File 1). Signaling pathway analysis was performed by mapping genes to KEGG pathways. As indicated in Online Fig. 5, TGF-β treatment dramatically altered these signaling pathways, such as collagen metabolism, migration, proliferation, paired-related homeobox family, nitrosative stress, oxidative stress, and contraction-relaxation, further supporting the role of Prrx1 S-nitrosylation in FMD.

### 3.12 AAV9-mediated exogenous expression of S-nitrosylation resistant Prrx1 prevents cardiac fibrosis and dysfunctions in mice with MI

**Fig. 6:**
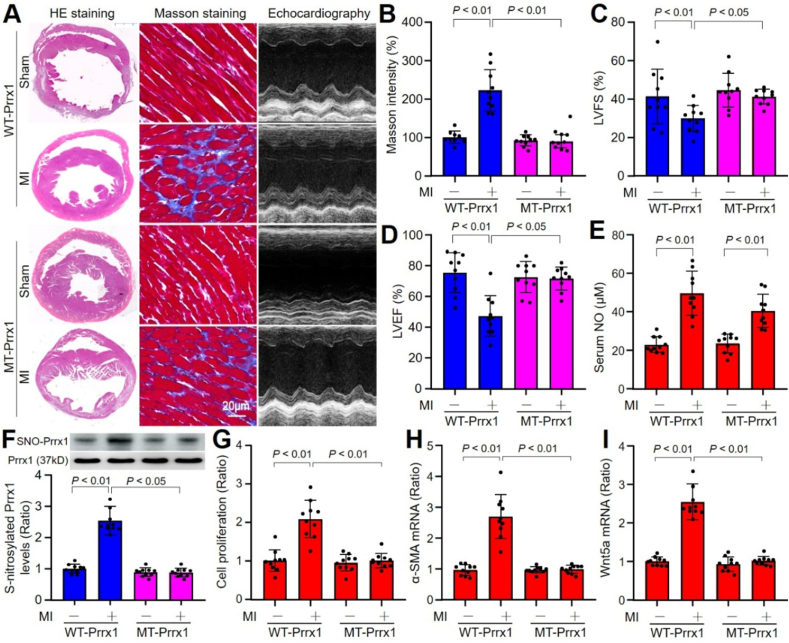
**Enforced expression of Prrx1-C209R improves cardiac remodeling in mice after myocardial infarction (MI)**. The experimental protocol was illustrated in Online Fig. 6A. (**A-D**) At the 28th postoperative day, hearts were harvested from mice to perform HE staining and Masson staining. Heart functions were measured before sacrificed using ultrasound. Representative images were shown in **A**. Collagen intensity in **B**, LVFS in **C**, and LVEF in **D** were calculated. (**E**) Serum NO concentrations. (**F–I**) Cardiac fibroblasts isolated from mice were subjected to determine Prrx1 S-nitrosylation using the biotin-switch method in **F**, cell proliferation using CCK8 in **G**, α-SMA mRNA in **H**, and Wnt5a mRNA in **I**. N = 10 per group. A one-way ANOVA followed by Tukey's HSD test was used to determine *P* value between two groups.

Cardiac fibrosis is a key factor to affect the recovery of heart functions in patients with ischemia heart diseases [37]. Knowing the role of Prrx1 S-nitrosylation in the regulation of FMD *in vitro*, we next investigated whether Prrx1 S-nitrosylation plays a key role in cardiac fibrosis *in vivo*. To this point, we infected mice with AAV9 expressing Prrx1-WT or Prrx1-MT (C209R), which is S-nitrosylation resistant to NO modification, and detected myocardial fibrosis using Masson staining at the 28th postoperative day (Online Fig. 6A and B). As indicated in Fig. 6A and B, MI significantly promoted cardiac fibrosis in mice infected with AAV9 expressing Prrx1-WT, but not in mice infected with AAV9 harboring Prrx1-MT.

We also examined heart functions at the 28th postoperative day by echocardiography and the representative images were shown in Fig. 6A. There were noticeably decreased LVEF and LVFS (Fig. 6C and D) in mice infected with AAV9 expressing Prrx1-WT, rather than mice infected with AAV9 expressing Prrx1-MT. Accordingly, there was increased NO productions and Prrx1 S-nitrosylation in mice expressing Prrx1-WT, but not Prrx1-MT (Fig. 6E and F). These data suggest that Prrx1 S-nitrosylation is vital to mediate cardiac remodeling during postischemia.

### 3.13 Prrx1 S-nitrosylation promotes FMD in mice with MI

We next determined the role of fibroblast-specific Prrx1 on cardiac fibrosis in mice. In vivo, gene expressions of α-SMA, Col I, and Col III were increased in hearts tissues of mice expressing with Prrx1-WT, rather than in mice with enforced expression of Prrx1-MT (Online Fig. 6C–E). We also measured these indices on cardiac fibroblasts isolated from mice. As indicated in Fig. 6G–I, their gene expressions were enhanced in cardiac fibroblasts of mice expressing with Prrx1-WT, but not in mice with enforced expression of Prrx1-MT, suggesting these effects are specific to cardiac fibroblasts, rather than other cardiac cells.

### 3.14 Fibroblast-specific Prrx1 gene knockout prevents cardiac fibrosis in mice following MI

**Fig. 7:**
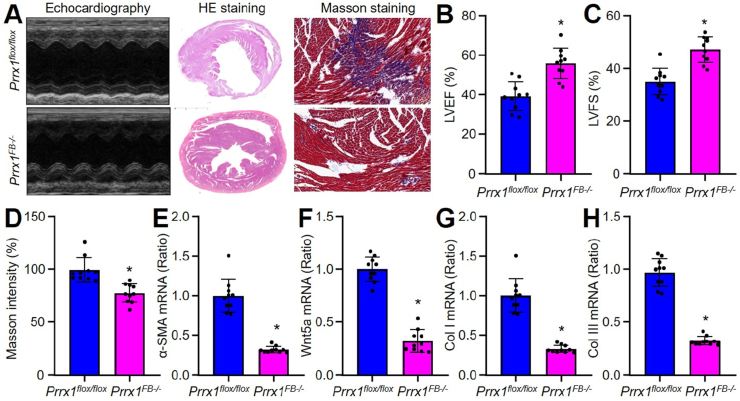
**Fibroblast-specific Prrx1 gene knockout prevents cardiac remodeling in mice following myocardial infarction (MI)**. The experimental protocol was illustrated in Online Fig. 8A. (**A-D**) At the 28th postoperative day, heart functions were measured before sacrificed using ultrasound. Hearts were harvested from mice to perform HE staining and Masson staining. Representative images were shown in **A**. LVEF in **B**, LVFS in **C**, collagen content in **D** were calculated. (**E-H**) Cardiac fibroblasts isolated from mice were subjected to gene expressions of α-SMA in **E**, Wnt5a in **F**, Col I in **G**, and Col III in **H** using quantitative PCR. N = 10 per group. An unpaired Student's *t*-test was used to determine *P* value.

Activated fibroblasts and myofibroblasts are the central cellular effectors in cardiac fibrosis, serving as the main source of matrix proteins. Besides, immune cells, vascular cells and cardiomyocytes may also acquire a fibrogenic phenotype under conditions of stress, activating fibroblast populations [38]. To exclude the contributions of other cells in Prrx1-mediaeted cardiac fibrosis, we generated fibroblast-specific Prrx1 gene knockout (*Prrx1*^*FB−/-*^) mice by crossing *Prrx1*^*flox/flox*^ mice with *Col1a2-iCre*^*ER*^ mice followed by MI surgery for 4 weeks (Online Fig. 7A and B and Online Fig. 8A). As indicated in Fig. 7A–D, compared to *Prrx1*^*flox/flox*^, there were improved heart functions and decreased cardiac fibrosis in *Prrx1*^*FB−/-*^ mice following MI. Both mRNA and protein levels of Wnt5a, α-SMA, Col I, and Col III were lower in the hearts of *Prrx1*^*FB−/-*^ mice, compared to the hearts of *Prrx1*^*flox/flox*^ after MI (Fig. 7E–H and Online Fig. 8B–F). These results further support that fibroblast Prrx1 is responsible for cardiac remodeling following MI.

**Fig. 8:**
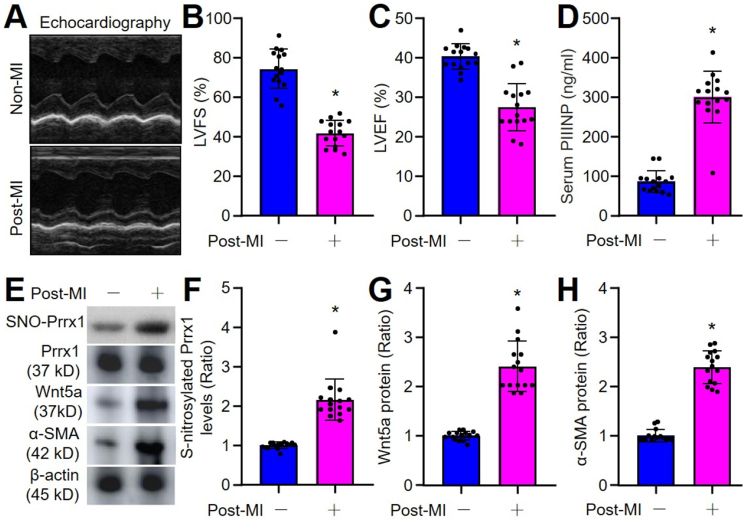
**Increased Prrx1 S-nitrosylation in patients with post myocardial infarction (MI)**. The demographic data of human subjects were presented in Online Table 1. (**A-D**) Heart functions were measured using ultrasound in **A**. LVEF in **B** and LVFS in **C** were calculated. Serum level of N-terminal propeptide procollagen type III (PIIINP) was measured using ELISA in **D**. (**E-H**) Leucocytes in peripheral blood isolated from patients. Total cell lysates were subjected to determine the levels of Prrx1 S-nitrosylation using the biotin-switch method, α-SMA and Wnt5a proteins using Western blot in **E**. Quantifications of Prrx1 S-nitrosylation in **F,** Wnt5a in **G**, and α-SMA in **H** were calculated. N = 15 per group. *P < 0.05 *vs.* Non-MI. An unpaired Student's *t*-test was used to determine *P* value.

### 3.15 Increased Prrx1 S-nitrosylation in patients with one-year post-MI

To provide translational perspectives of this study, we finally conducted a pilot experiment in patients with acute MI history. The demographic data of human subjects were presented in Online Table 1. As presented, there were impaired heart functions (Fig. 8A–C), increased serum levels of N-terminal propeptide procollagen type III (Fig. 8D), a biomarker of cardiac fibrosis [39], and higher levels of serum NO (Online Table 1), in patients with one-year post-MI, compared to patients without MI. Moreover, the levels of Prrx1 S-nitrosylation, Wnt5a protein, and α-SMA protein were increased in peripheral blood leucocytes isolated from in patients with one-year post-MI (Fig. 8E–H). Although the small-sample clinical investigation did not establish the cause-effect relation between Prrx1 and cardiac fibrosis, it still uncovers the important role of Prrx1 in the post-ischemic recovery of heart function.

## 4 Discussion

In the present study, we provided the first evidence that NO-mediated S-nitrosylation upregulates Prrx1 transcriptional activity to activate Wnt5a signaling, which induces FMD of cardiac fibroblasts. As a result, this contributes to cardiac remodeling and the delayed recovery of heart functions after MI (Online Fig. 9). This novel mechanism not only uncovers a molecular mechanism by which Prrx1 is activated in cardiac fibroblasts, but also provides a novel target for exploring new drugs to improve the prognosis of ischemic heart disease.

The major finding of this study is that Prrx1 activity is regulated by S-nitrosylation. Protein post-translational modifications, including phosphorylation, ubiquitination, glycation, etc., are important to determine protein functions and play key roles in many cellular processes [40]. This study provides further evidence to support the proposal that Prrx1 function is tightly controlled by S-nitrosylation as followings. First, human recombinant Prrx1 protein is S-nitrosylated by structurally unrelated NO donors. Second, Prrx1 S-nitrosylation is detectable in OGD/TGF-β-treated cardiac fibroblast *in vitro* and mice following MI *in vivo*. Further, we identified that the S-nitrosylation site within Prrx1 protein is C207 in human protein using mass spectrometry or site-directed mutagenesis. This study provides new evidence to support the proposal that Prrx1 function is greatly related to Prrx1 S-nitrosylation.

Another discovery of this project is that post MI-induced cardiac fibrosis is Prrx1 S-nitrosylation dependent. Myocardial fibrosis, the expansion of the cardiac interstitium through deposition of extracellular matrix proteins, is a common pathophysiologic companion of many different myocardial conditions. Cardiac fibrosis is an important pathological process contributing to the pathogenesis of cardiac remodeling after MI, which is a transition from an early inflammatory phase to fibrotic granulation and maturation stage of cardiac remodeling [6]. As a matter of fact, myocardial fibrosis is the endpoints of cell differentiation, activation, and proliferation of cardiac fibroblasts [41,42]. Our study explains the molecular mechanism by how MI induces FMD of cardiac fibroblasts in myocardial remodeling through Prrx1 S-nitrosylation.

As concerned about why Prrx1 gene expression is elevated in cardiac fibroblasts after MI. An underlined mechanism is through the production of TGF-β, which is a key factor contributing to cardiac fibrosis in heart diseases [43,44]. Here we reported that TGF-β can induce Prrx1 S-nitrosylation in cardiac fibroblasts and further demonstrated that enforced expression of S-nitrosylation-resistant Prrx1, which is resistant to S-nitrosylation, has a marked protective effect against cardiac fibrosis and heart dysfunctions following MI. These observations were replicated in OGD-treated cardiac fibrosis. These findings suggest a critical role for Prrx1 S-nitrosylation in cardiac remodeling and the recovery of heart functions after MI.

On the other hand, the role of FMD in cardiac remodeling after MI is very controversial. Fibrosis may reflect activation of reparative or maladaptive processes. As reported by Yang K et al., AlkB homolog 5 (ALKBH5) induces FMD during hypoxia to protect against cardiac rupture, which is a dramatic and potentially lethal mechanical complication of MI [45]. They demonstrated that fibroblast ALKBH5 positively regulates post-MI healing to prevent cardiac rupture. We propose that this discrepancy may be explained by different timepoints after MI. They observed that ALKBH5-mediated FMD was increased from day 1, peaked at day 5, and returned to baseline level at day 28 after MI. Generally, there is high risk of cardiac rupture within the 1st week once MI occurs. In this study, we reported that Prrx1-mediated FMD contributes to cardiac fibrosis at the 28th post-MI day, consistent with other reports [21,46,47]. Importantly, we observed that cardiac fibrosis was limited and the recovery of heart functions was promoted by NAC at the 28th post-MI day.

There are some limitations of this study. First, the pharmacological mechanisms of NAC are extensive, in which the SH group potentially reacts with NO, which is required for both 3-nitrotyrosine and *S*-nitrosylation. Though we and other groups used NAC as anti-S-nitrosylation reagent [[48], [49], [50], [51], [52]], it is hard to differ whether the pharmacological effects of NAC depend on 3-nitrotyrosine or *S*-nitrosylation. Second, we reported that S-nitrosylation occurs under OGD conditions in cells, 28 days after MI in mice or patients with one-year post-MI. On the one hand, NO synthesis requires oxygen, since it supplies half of the molecule. As reported by literatures, metabolic reprogramming from glucose to fatty acid plays crucial roles in MI repair [[53], [54], [55]]. Oxygen is required for both glucose oxidation to fatty acid oxidation. Therefore, we speculated that oxygen supply is possibly reversed because we observed that NO productions were increased at these time-points.

In summary (Online Fig. 9), the present study proposes a role of Prrx1 S-nitrosylation in cardiac remodeling in response to ischemic injury. When MI happens, due to ischemia, Prrx1 is activated by TGF-β through S-nitrosylation to enhance Wnt5a signaling, which induces FMD. In this way, MI promotes cardiac fibrosis to delay the recovery of heart functions. In future, protective strategies on cardiac remodeling by inhibition of Prrx1 S-nitrosylation may improve the prognosis of patients with ischemia heart disease liking MI.

## CRediT authorship contribution statement

**Dashuai Wang:** Validation, Supervision, Software, Resources, Project administration, Methodology, Investigation, Funding acquisition, Formal analysis, Data curation. **Shaoxuan Zhou:** Methodology. **Yajing Tang:** Methodology. **Zhenxing Liang:** Methodology. **Xinyi Yu:** Methodology. **Gongcheng Huang:** Methodology. **Chen Huang:** Methodology. **Shuangxi Wang:** Writing – review & editing, Writing – original draft, Visualization, Validation, Supervision, Software, Resources, Project administration, Methodology, Investigation, Funding acquisition, Formal analysis, Data curation, Conceptualization. **Hai Liu:** Writing – review & editing, Writing – original draft, Visualization, Validation, Supervision, Software, Resources, Project administration, Methodology, Investigation, Funding acquisition, Formal analysis, Data curation, Conceptualization.

## Ethical approval and consent to participate consent for publication

The animal protocol was reviewed and approved by the Animal Care and Use Committee, Zhengzhou University. The clinical study protocol was approved by the Ethical Committee of The First Affiliated Hospital of Zhengzhou University, and informed consent was obtained from each human subject.

## Availability of data and materials

We are willing to make all data, analytic methods, and study materials available to other researchers upon reasonable request.

## Funding

This study was supported by the 10.13039/501100001809National Natural Science Foundation of China (No. 81500260) and the Scientific and Technological Research Project of Henan Province (No. 242102310156).

## Declaration of competing interest

The authors declare that they have no competing interests.

## Data Availability

Data will be made available on request.
