# Supplementary material for: S-nitrosylation of paired-related homeobox 1 promotes cardiac remodeling following myocardial infarction
Source: Redox Biol. 2025 Oct 30;87:103887. doi: 10.1016/j.redox.2025.103887 (PMC12628024; doi:10.1016/j.redox.2025.103887)
Supplement: Multimedia component 2 [file mmc2.docx]

**Online Materials and Methods**

**Materials**

N-acetyl-cysteine (NAC), carboxy-PTIO, and sodium nitroprusside (SNP) were bought from Tocris Bioscience. Dihydroethidium (DHE) and diaminofluorescein (DAF) were purchased from Calbiochemical Company (USA). Recombinant human Prrx1 protein and biotin switch assay kit (S-Nitrosylation) (ab236207) was obtained from Abcam Company (San Francisco, USA). Primary antibodies against His, Prrx1, α‑SMA, Col I, Col III, and β-actin were purchased from Cell Signaling Transduction Company or Santa Cruz Company. Protein A/G plus-agarose and secondary antibody were obtained from Santa Cruz Biotechnology Inc. Commercial kits for determinations of glucose, cholesterol, nitric oxide (NO), and triglyceride, LDL-C, and HDL-C were purchased from Jian-Cheng Bioengineering Institute (Nanjing, China). All drug concentrations are expressed as working concentrations in the buffer.

**Animals and experimental protocols**

Mice, 8-12 weeks of age, were obtained from Beijing Huafukang Animal Experimental Center. Mice were housed in temperature-controlled cages with a 12-h light-dark cycle and given free access to water and a regular diet. This animal study was carried out in strict accordance with the recommendations in the Guide for the Care and Use of Laboratory Animals of the National Institutes of Health. The animal protocol was reviewed and approved by the Animal Care and Use Committee, Zhengzhou University.

**Myocardial infarction (MI)**

MI surgery was performed as previously described^1^.

1. Sterilize surgical instruments with a dry bead sterilizer (Germinator 500).

2. All mice (aged 8-12 weeks) were anesthetized with 2-3% isoflurane inhalation in an inducing chamber.

3. Once anesthetized, the mouse is removed from the inducing chamber to the surgical board, immobilized with tape, and continuously anesthetized with 2% isoflurane via coaxial breathing apparatus but not ventilated.

4. Remove the fur with a standard depilatory (e.g., Nair) and clean the skin with water and then betadine and alcohol pads. In order to perform this procedure more efficiently, the step of fur-removing could be done earlier.

5. Two small incisions (0.5 cm long) are made on the left and right chest skin with the scissors to expose the 3rd intercostal space.

6. Echocardiography is performed using a VEVO 2100 imaging system (Visual Sonics Inc., Toronto, Canada) with a 30 MHz phased array transducer and a frame rate of 235/s. The echocardiography probe (MS-400) is placed perpendicular to the sagittal plane of the chest within the 3rd intercostal space, imaging the left ventricle (LV) short axis.

7. A small straight needle (0.2 mm in diameter) was inserted at the costal angle of the superior margin of the 3^rd^ rib in the left chest. Under the guide of ultrasound, the heart is punctured in the inferior of left anterior descending coronary artery (LADCA) by a 8-0 silk suture attached to the needle. The needle is coming out of skin from the right chest.

8. Then, the needle is inserted back from the right to of the left. When the needle passes through the heart, it goes through above LADCA under ultrasound and came out the skin from the same site in the left chest.

9. Once a loose knot is made, the needle is inserted back from the left to the right in the chest. The LADCA is now located inside of the knot.

10. Ligation of LADCA by pulling the two ends of the suture carefully. The ischemia was confirmed by the elevation of ST segment recorded by the echocardiography imaging system during the surgery. The knot is readily visible under ultrasound.

11. The mouse is then allowed to breathe room air and monitored on a heating blanket during the recovery period, which is generally complete within 3-5 min.

12. The sham group undergoes the same surgical procedures except that the LADCA is not occluded.

13. One dose of buprenorphine (0.1 mg/kg) is administered subcutaneously (s.c.) immediately after the incision is closed.

The experimenters were blind to treatment grouping information during the experimental procedures and their quantifications. Welfare-related assessments, measurements and interventions were carried out before, during and after the experiment.

During the chronic period following MI surgery, all animals were housed in a controlled environment with adequate amounts of food and water provided in individually ventilated cages with same size and wood shavings as bedding material. All animals were kept in a specific pathogen-free facility. At the end of the study, animals that underwent MI surgery were humanely killed by CO_2_ euthanasia and the condition of death was confirmed by cervical dislocation.

**Echocardiography**

Echocardiography was performed as described previously^2^. Echocardiography was performed with a Vevo2100 Imaging System (Visual Sonics Inc.) with a MS-400 ultrasound transducer. After anesthetization (2% inhaled isoflurane), the mouse’s left ventricular (LV) was assessed in both parasternal short-axis and long-axis view. The end-systole and end-diastole were defined as the phase in which the smallest or largest LV area was obtained, respectively. All echocardiography was performed by the same investigator who was blinded to the experimental groups.

**Masson’s trichrome staining**

As described previously^3^, heart samples were fixed in 4% PFA and then embedded in paraffin. Five μm-thick sections were subjected to Masson’s trichrome staining following a standard procedure. Images of the left ventricular area of each section were taken (200X magnification) with Spot Insight camera. Image J Software (National Institutes of Health) was used to quantify fibrotic region in each section. The percentage of fibrosis was measured as fibrosis areas/total left ventricular areas X 100%.

**Hematoxylin-eosin (HE) staining**

As described previously^4^, slides with section were placed in a metal staining rack and immersed in the filtered Harris Hematoxylin for 10 seconds. Then, the sections were incubated in EOSIN stain for 30 seconds. Dehydration was performed in ascending alcohol solutions (50%, 70%, 80%, 95% X 2, 100% X 2) followed by clearance with xylene (3-4 X) in in Columbia staining jars. The slides were mount using Permount (xylene based).

**Generations of DNA constructs**

WT-Prrx1 cDNA were purchased from Origene Company. Cysteine residue was replaced with arginine by using the QuikChange kit (Stratagene), according to the manufacturer's instructions. The mutation was confirmed by DNA sequencing. The virus construction compassing WT-Prrx1 or MT-Prrx1 (C207R or C209R) was generated using the AdMax (Microbix) and pSilencer™ adeno 1.0-CMV (Ambion) systems. Viruses were packaged and amplified in HEK293A cells and purified using CsCl_2_ banding followed by dialysis against 10 mM Tris-buffered saline with 10% glycerol. Titering was performed on HEK293 cells using the Adeno-X Rapid Titer kit (BD Biosciences Clontech, Palo Alto, CA, USA) according to the manufacturer’s instructions.

**Generation of shRNA construct**

Based on the protocol from Signaling Gateway, the shRNA cassette containing target sequence of Wnt5a was designed. The cassette was subcloned into pEN-hH1c vector as described previously^5^. And then the pEN-hH1c vector containing the Wnt5a shRNA cassette was combined with an attR-containing vector pDSL-hpUP in an LR recombination reaction. The recombinant constructs pDSL-hpUP-Wnt5a-shRNA was confirmed by DNA sequence analysis. The sequence of Wnt5a shRNA is CCGGGCTAATTCTTGGTGGTCT CTACTCGAGTAGAGACCACCAAGAATTAGCTTTTTG. The sequence of negative control shRNA is TTCTCCGAACGTGTCACGT. The adenovirus was produced by transiently transfecting HEK293T cells using SuperFect transfection reagent (Qiagen, USA) with three packing plasmid system (pGag/Pol, pRev, and pVSV-G). The virus-containing supernatant was collected 72 hours after transfection, and filtered through 0.45 mm filters (Millipore, USA), and stored at -80°C. The titer of the viral vectors was determined by TCID50 (Tissue culture infective dose) method.

**Animal experimental protocols**

In the first part of the animal study (Online Figure 1A), wildtype mice were treated with NAC (50 mg/kg/day) for 6 consecutive weeks followed by the permanent ligation of LADCA. At the 28^th^ post operative day, echocardiography was performed to determine heart functions before scarified. At the end of experiment, heart tissues were harvested.

In the second part of the animal study (Online Figure 6A), mice were injected with AAV9 expressing Prrx1 (*WT*, C209R) per 6 weeks. Two weeks later, mice received the surgery of LADCA permanent ligation. At the 28^th^ post operative day, echocardiography was performed to determine heart functions before scarified. At the end of experiment, heart tissues were harvested.

In the third part of the animal study (Online Figure 8A), *Prrx1^flox/flox^* and *Prrx1^FB-/-^* mice received the surgery of LADCA permanent ligation. At the 28^th^ post operative day, echocardiography was performed to determine heart functions before scarified. At the end of experiment, heart tissues were harvested.

Isolation and culture of cardiac fibroblasts from mice

Fibroblasts were isolated from mice hearts as previously described^4^. Cardiac fibroblasts were cultured in Dulbecco’s modified Eagle’s medium (DMEM, HyClone) supplemented with 10% fetal bovine serum in a humidified atmosphere with 5% CO_2_ at 37°C. Only cells with no more than three passages were used in this study.

**Viral infection to cells and animals**

Cells were infected with adenovirus overnight in antibiotics-free medium supplemented with 2% FBS. The cells were then washed and incubated in fresh medium for an additional 12-hour before experiments. For infecting animals, AAV8 was injected via tail vein in 100 µl of PBS containing 7.6 X 10^8^ IFUs of loaded virus per rat as described previously^6-8^. The concentration of DNA was 10 mg/kg.

**Cell Counting Kit 8 (CCK8) assay**

The cells were seeded into 96-well plates at a concentration of 2000 cells per well in 3 replicate wells. CCK-8 solution (Sangon Biotech) was added to each well. After incubation in a 5% CO_2_ atmosphere at 37^∘^C for 2 hours, the mixture was shaken for 1 minute on a shaker in the dark. Then, absorbance at 450 nm was measured using a microplate reader. The absorbance value was used as the ordinate and the interval time was used as the abscissa. The value of CCK8 intensity in control group is set as 1. Other groups were normalized as control group.

**Protein S-nitrosylation assay**

Proteins were extracted according to the manufacturer's specification S-Nitrosylated Protein Detection Assay Kit (Cayman, USA) which is based on the “Biotin-switch” method. Using this method, protein free thiols were blocked with a blocking agent, and then any S-Nitrosothiols in samples were reduced to yield free thiol(s), and subsequently covalently labeled with biotin. After the quantification of protein concentration, equal amounts proteins of control and flavone-treated cells were purified by streptavidin magnetic beads. After purification, streptavidin magnetic beads were washed by water (pH 6.7) for six times, and then S-nitrosylated proteins were separated from streptavidin magnetic beads by 1:5 loading buffer. The S-nitrosylated proteins were detected by western blot.

**Mass spectrometry (MS)**

To identify the S-nitrosylation site, HEK293T cells stably expressing HA-tagged Prrx1 were treated with 2 mM SNP for 2 hours before harvest and then lysed. The lysates were purified using anti-HA-agarose beads. The pellet was then resolved on SDS-PAGE and stained with Coomassie blue staining. The band corresponding to HA-Prrx1 was excised and sent for mass spectrometry analysis.

**RNA extraction, sequencing, and data processing**

Total RNA was isolated using a TRIzol-based (Invitrogen) RNA isolation protocol. RNA was quantified by Nanodrop (Agilent Technologies), and RNA and mRNA quality were verified using an Agilent 2100 Bioanalyzer (Agilent Technologies). Samples required 260/280 ratios of more than 1.8, and sample RNA integrity numbers of more than 9 for inclusion. Sequencing data were acquired from the Gene Expression Omnibus (accession no. GSE166676). The Seurat package (Version 3.2.0) in R software (Version 4.2.1) was used for cross-sample adjustment, processing, and quality control. Total RNA from 3 samples was extracted. Briefly, the mRNA with polyA in the total RNA was enriched by Oligo-dT magnetic beads. The intact mRNA was then fragmented using an ultrasound machine. The segmented RNA was used as an input to directly construct a conventional transcriptome sequencing library (RNA-seq). The conventional sequencing library was constructed according to the transcriptome library construction process. Illumina Hiseq X Ten was used for high-throughput sequencing of the library. The mRNA base database and Circ2Traits were used to annotate the identified mRNA. Then, DESeq2 software (v1.14.1) was used for data standardization and differentially expressed mRNA screening (log_2_FC ≥ 1.5, p-value ≤ 0.05). Enrichment analysis for the functions of differentially expressed genes (DEGs) was conducted using the Gene Ontology (GO) and Kyoto Encyclopedia of Genes and Genomes (KEGG) functions in the Cluster Profiler in R software; enrichment in GO terms and KEGG pathways were calculated based on hypergeometric distribution. The functions with p-value < 0.05 were considered significantly different.

**Plasmid transfection into HEK293 and Reporter Assays**

The plasmid constructs (WT-Prrx1 or MT-Prrx1) were co-transfected in HEK293 cells with the pCMV β-gal plasmid by using lipofectamine 2000 (Invitrogen) as described previously^9^. Cells were harvested 48 hours after transfection, and luciferase and β-galactosidase activities measured.

**Western blot**

Cells or tissues were homogenized on ice in cell-lysis buffer containing 20 mM Tris-HCl (pH 7.5), 150 mM NaCl, 1 mM Na_2_EDTA, 1 mM EGTA, 1% Triton, 2.5 mM sodium pyrophosphate, 1 mM beta-glycerophosphate, 1 mM Na_3_VO_4_, 1 µg/ml leupeptin, and 1 mM PMSF. Protein samples were solubilized in SDS sample buffer, and 20 µg of protein was separated by SDS-PAGE using 8-10% polyacrylamide gels, transferred to nitrocellulose membranes. Entire sheets of hybond-ECL membranes containing transferred proteins were incubated firstly in 5% non-fat dry milk for 2 hours to block nonspecific binding of antibodies, followed by overnight incubation in primary antibodies diluted 1:1000 at 4°C. The membranes were then washed 3 times with TBST and incubated for 1 hour with second antibody diluted 1:5000 at room temperature. Bound antibodies were detected with ECL-enhanced chemiluminescence (Amersham Biosciences) according to the manufacturer's protocols. Hyperfilm-ECL exposed to membranes for 1 minute was developed in an X-ray film processor. Band intensity (area X density) was measured by densitometry (model GS-700, Imaging Densitometer; Bio-Rad). Background intensity was subtracted from all calculated areas and we used the ratio of control group as 1 as described previously^10^.

**Immunofluorescence (IFC)**

After treatment, cells on sterile glass cover slips were rinsed by cold PBS and then fixed by incubation with 10% formalin in PBS for 10 minutes. Block cells by 5% BSA for 30 minutes. Incubate cells with primary antibody for 1 hour at room temperature or overnight at 4^o^C. After washing, incubate with fluorescence-conjugated secondary antibody for 45 minutes. Take picture in fluorescence microscope.

**Detection of ROS**

ROS levels were measured using the DHE fluorescence as described previously^9^. Briefly, cells or tissues were incubated with DHE (10 µM) for 30 min, homogenized, and subjected to methanol extraction. HPLC was performed using a C-18 column (mobile phase: gradient of acetonitrile and 0.1% trifluoroacetic acid) to separate and quantify oxyethidium (product of DHE and O_2_^-^) and ethidium (a product of DHE auto-oxidation). ROS level was determined by conversion of DHE into oxyethidine.

**Detection of NO**

NO production in cells or tissues was detected using the fluorescent probe DAF as described previously^11^. Briefly, before the end of treatment, 10 µM DAF was added to the medium and incubated for 30 min at 37°C, then washing with PBS twice. The DAF fluorescent intensity was recorded by fluorescent reader at the wave of excitation (485 nm) and emission (545 nm). Serum NO was assayed by using commercial kits as recommend by the provider through the Gries method as described by us previously^12^.

**Patients and sample processing**

We finally collected a total of 15 patients who underwent acute MI with coronary angiography at Department of Cardiovascular Surgery, The First Affiliated Hospital of Zhengzhou University. These patients were followed up at least 1-year. 15 patients who did not undergo acute MI were recruited as control. The demographic data for these individuals were listed in Online Table 1. Leucocytes were isolated from blood and subjected to biochemical analysis and western blot analysis. Informed consent was obtained from all participants. The procedures were in accordance with the ethical standards of the responsible committee on human experimentation or with the Helsinki Declaration of 1975. These protocols complied with the Management Rules of the Chinese Ministry of Health and were approved by the Ethical Committee of The First Affiliated Hospital of Zhengzhou University (KYLL-2020-7258), and informed consent was obtained from each human subject.

**Enzyme-linked immunosorbent assay (ELISA)**

Serum N-terminal propeptide procollagen type III (PIIINP) levels were measured using an ELISA with laboratory kits (Cloud-Clone Corp., Katy, TX, 77494, USA). Serum PIIINP levels in the study sample were compared with the values measured in the control group. PIINP concentration in the control group was 7.2 [6.8-7.5] ng/ml.

**Statistical analysis**

All quantitative results were expressed as mean ± SD. The normal distribution of data was tested by the Kolmogorov-Smirnov test before statistical comparisons, and the normality/equal variance was tested to determine whether ANOVA was appropriate. All data were analyzed with a 1-way ANOVA followed by Tukey’s HSD test or Dunnett's test. An unpaired Student's *t* test was used for comparison between two groups. Statistical analysis was conducted using IBM SPSS statistics 20.0 (IBM Corp., Armonk, NY, USA), and *P* < 0.05 was considered significant.

**References**

1. Sun Q, Wang KK, Pan M, Zhou JP, Qiu XT, Wang ZY, Yang Z, Chen Y, Shen H, Gu QL, Fang LH, Zhang GG and Bai YP. A minimally invasive approach to induce myocardial infarction in mice without thoracotomy. *J Cell Mol Med*. 2018;22:5208-5219.

2. Yang J, Liu X, Jiang G, Chen Y, Zhang Y and Zhang M. Two-dimensional strain technique to detect the function of coronary collateral circulation. *Coron Artery Dis*. 2012;23:188-94.

3. Bai WW, Xing YF, Wang B, Lu XT, Wang YB, Sun YY, Liu XQ, Guo T and Zhao YX. Tongxinluo Improves Cardiac Function and Ameliorates Ventricular Remodeling in Mice Model of Myocardial Infarction through Enhancing Angiogenesis. *Evid Based Complement Alternat Med*. 2013;2013:813247.

4. Bai WW, Tang ZY, Shan TC, Jing XJ, Li P, Qin WD, Song P, Wang B, Xu J, Liu Z, Yu HY, Ma ZM, Wang SX, Liu C and Guo T. Up-regulation of paired-related homeobox 2 promotes cardiac fibrosis in mice following myocardial infarction by targeting of Wnt5a. *J Cell Mol Med*. 2020;24:2319-2329.

5. Godec J, Cowley GS, Barnitz RA, Root DE, Sharpe AH and Haining WN. Inducible RNAi in vivo reveals that the transcription factor BATF is required to initiate but not maintain CD8+ T-cell effector differentiation. *Proc Natl Acad Sci U S A*. 2015;112:512-7.

6. Maegdefessel L, Azuma J, Toh R, Merk DR, Deng A, Chin JT, Raaz U, Schoelmerich AM, Raiesdana A, Leeper NJ, McConnell MV, Dalman RL, Spin JM and Tsao PS. Inhibition of microRNA-29b reduces murine abdominal aortic aneurysm development. *J Clin Invest*. 2012;122:497-506.

7. Maegdefessel L, Azuma J, Toh R, Deng A, Merk DR, Raiesdana A, Leeper NJ, Raaz U, Schoelmerich AM, McConnell MV, Dalman RL, Spin JM and Tsao PS. MicroRNA-21 blocks abdominal aortic aneurysm development and nicotine-augmented expansion. *Sci Transl Med*. 2012;4:122ra22.

8. Wang J, Guo T, Peng QS, Yue SW and Wang SX. Berberine via suppression of transient receptor potential vanilloid 4 channel improves vascular stiffness in mice. *J Cell Mol Med*. 2015;19:2607-16.

9. Wang S, Xu J, Song P, Wu Y, Zhang J, Chul Choi H and Zou MH. Acute inhibition of guanosine triphosphate cyclohydrolase 1 uncouples endothelial nitric oxide synthase and elevates blood pressure. *Hypertension*. 2008;52:484-90.

10. Wang S, Xu J, Song P, Viollet B and Zou MH. In vivo activation of AMP-activated protein kinase attenuates diabetes-enhanced degradation of GTP cyclohydrolase I. *Diabetes*. 2009;58:1893-901.

11. Thomas S, Kotamraju S, Zielonka J, Harder DR and Kalyanaraman B. Hydrogen peroxide induces nitric oxide and proteosome activity in endothelial cells: a bell-shaped signaling response. *Free Radic Biol Med*. 2007;42:1049-61.

12. Li P, Yin YL, Guo T, Sun XY, Ma H, Zhu ML, Zhao FR, Xu P, Chen Y, Wan GR, Jiang F, Peng QS, Liu C, Liu LY and Wang SX. Inhibition of Aberrant MicroRNA-133a Expression in Endothelial Cells by Statin Prevents Endothelial Dysfunction by Targeting GTP Cyclohydrolase 1 in Vivo. *Circulation*. 2016;134:1752-1765.


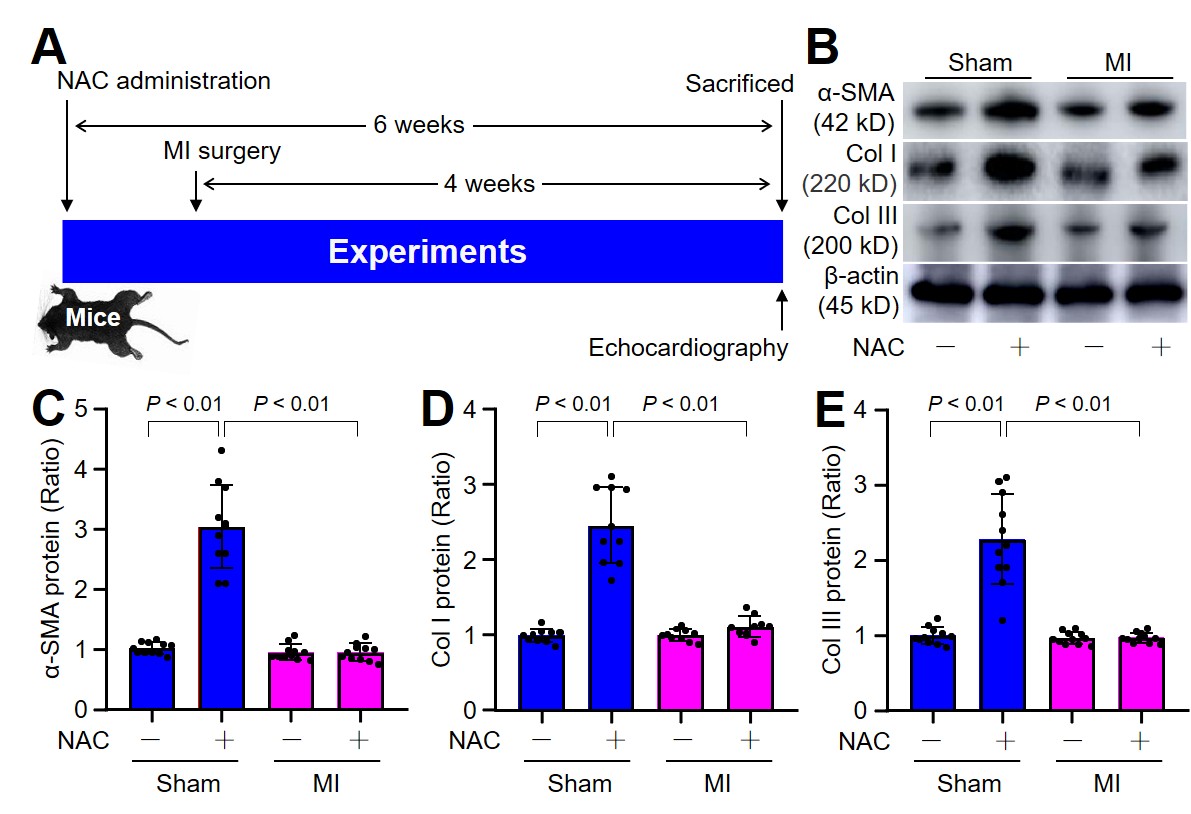


**Online Figure 1. Myocardial infarction (MI) induces fibroblast-to-myofibroblast** **differentiation in mice through S-nitrosylation in mice.** (**A**) Mice were treated with NAC (50 mg/kg/day) for 6 consecutive weeks followed by the permanent ligation of LADCA. At the 28^th^ post operative day, echocardiography was performed to determine heart functions before scarified. At the end of experiment, heart tissues were harvested. (**B-E**) Homogenates of heart were subjected to perform western blot analysis in **B**. Quantifications of α‑SMA in **C**, Col I in **D**, and Col III in **E** were performed. N = 10 per group. A one-way ANOVA followed by Tukey’s HSD test was used to determine *P* value between two groups.


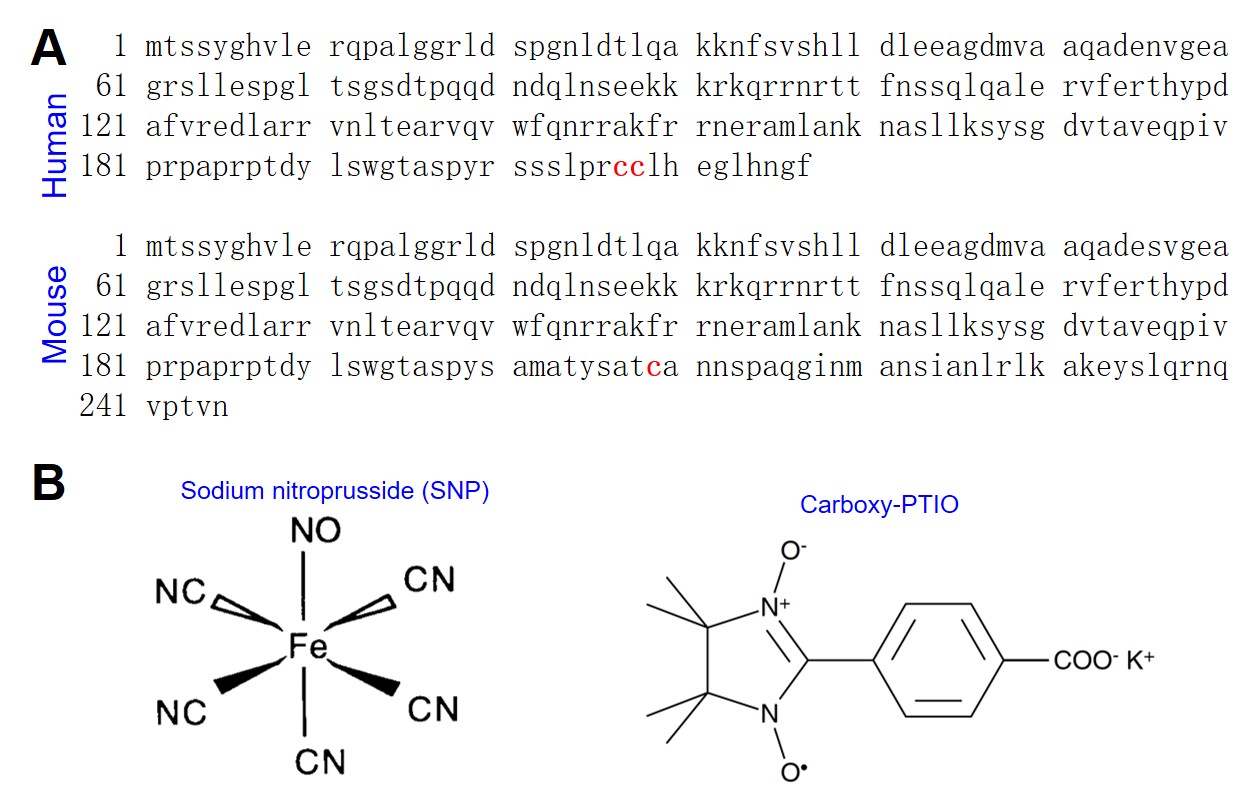


**Online Figure 2. Amino acid sequence of human Prrx1 protein and chemical sstructures of nitric oxide donors**. (**A**) Amino acid sequence of human Prrx1 protein and mouse Prrx1 protein. (**B**) Chemical structures of carboxy-PTIO and sodium nitroprusside (SNP).


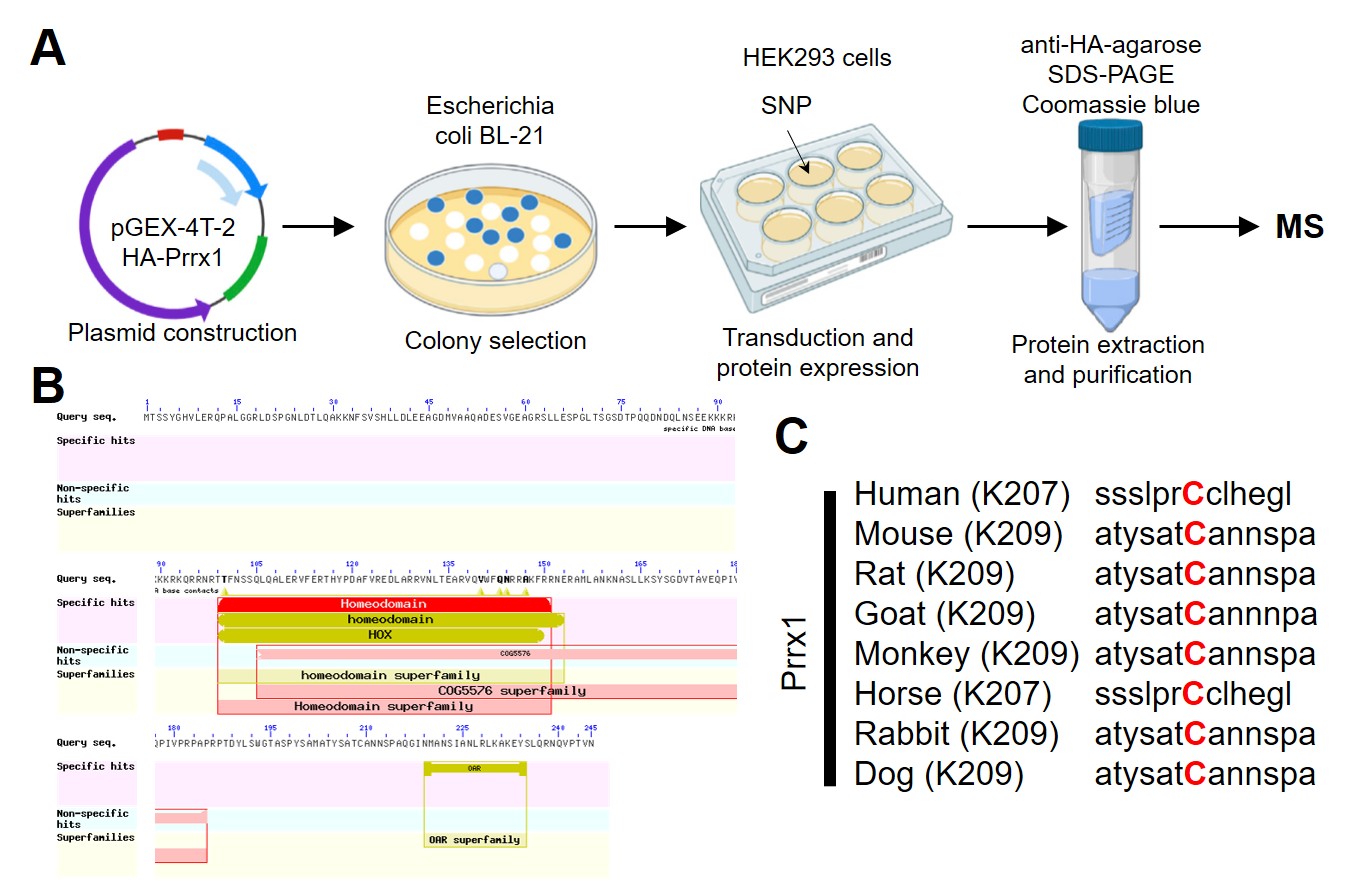


**Online Figure 3. Identification human Prrx1 S-nitrosylation by mass spectrometry (MS).** (**A**) Bacterial expression construct containing the full-length human Prrx1 gene was transformed into *Escherichia coli* BL-21. HEK293 cells stably expressing HA-tagged Prrx1 were treated with 1 mM sodium nitroprusside for 2 hours before harvest and then lysed. The lysates were purified using anti-HA-agarose beads. The pellet was then resolved on SDS-PAGE and stained with Coomassie blue staining. The band corresponding to HA-Prrx1 was excised and sent for MS analysis. (**B**) Domain analyses used an array of bioinformatics tools including SMART (http://smart.embl-heidelberg.de/), TMpred (http://www.ch.embnet.org/software/TMPRED_form.html), Coils (http://www.ch.embnet.org/software/COILS_form.html), and SignalP v4.1 (http://www.cbs.dtu.dk/services/SignalP/) for the presence of functional or structural domains and presence or absence of Sec-dependent secretion signals. (**C**) Sequence alignment of Prrx1 protein in various species.


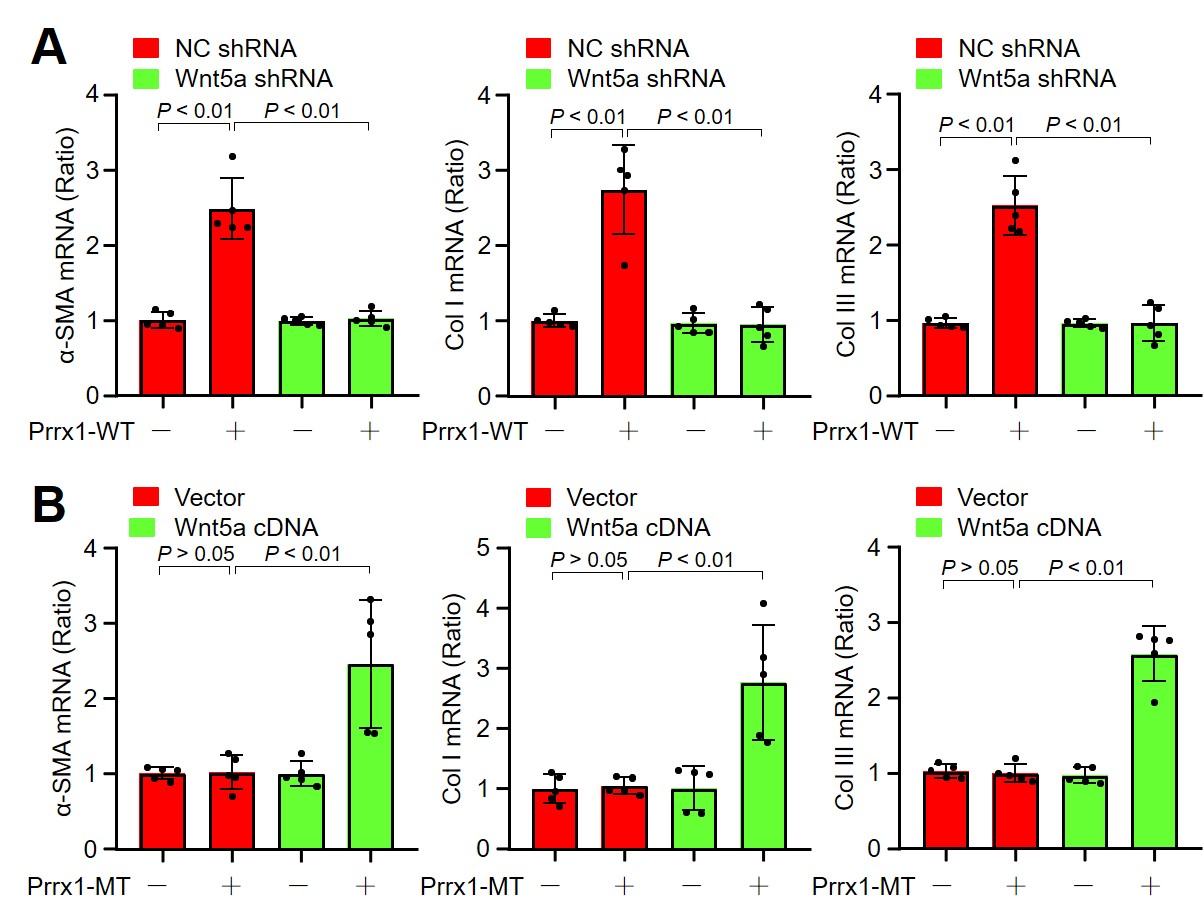


**Online Figure 4. TGF-β-induced Prrx1-mediated FMD is Wnt5a dependent in cardiac fibroblasts**. (**A**) Cardiac fibroblasts were infected with adenovirus expressing Prrx1-WT plus negative control (NC) or Wnt5a shRNA for 48 hours followed by TGF-β (10 ng/ml) treatment for 24 hours. (**B**) Cardiac fibroblasts were infected with adenovirus expressing Prrx1-MT (C207R) plus Wnt5a cDNA for 48 hours followed by TGF-β (10 ng/ml) treatment for 24 hours. Total RNA was extracted to assay the gene expressions of α‑SMA, Col I, and Col III using real-time PCR. N = 5 per group. A one-way ANOVA followed by Tukey’s HSD test was used to determine *P* value between two groups.


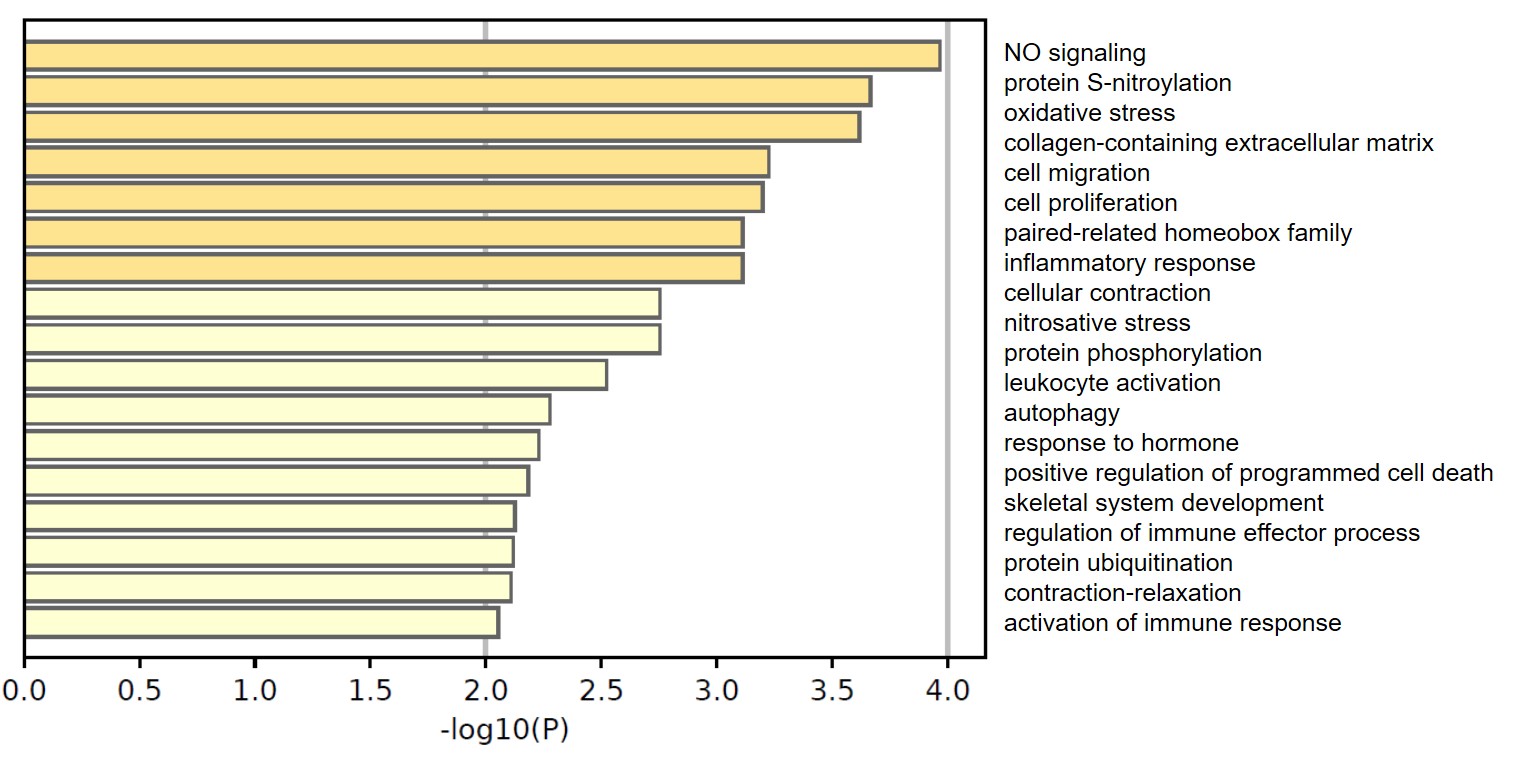


**Online Figure 5. Responses of cardiac fibroblasts to** **transforming growth factor beta (TGF-β).** Cardiac fibroblasts were incubated with TGF-β (10 ng/ml) for 24 hours. Differentially expressed genes were analyzed by RNA sequencing. Signaling pathway analysis was performed by mapping genes to KEGG pathways. N = 3 per group. The p-value denotes the significance of the pathway correlated to the conditions. The source of data was provided in Supplementary File 1.


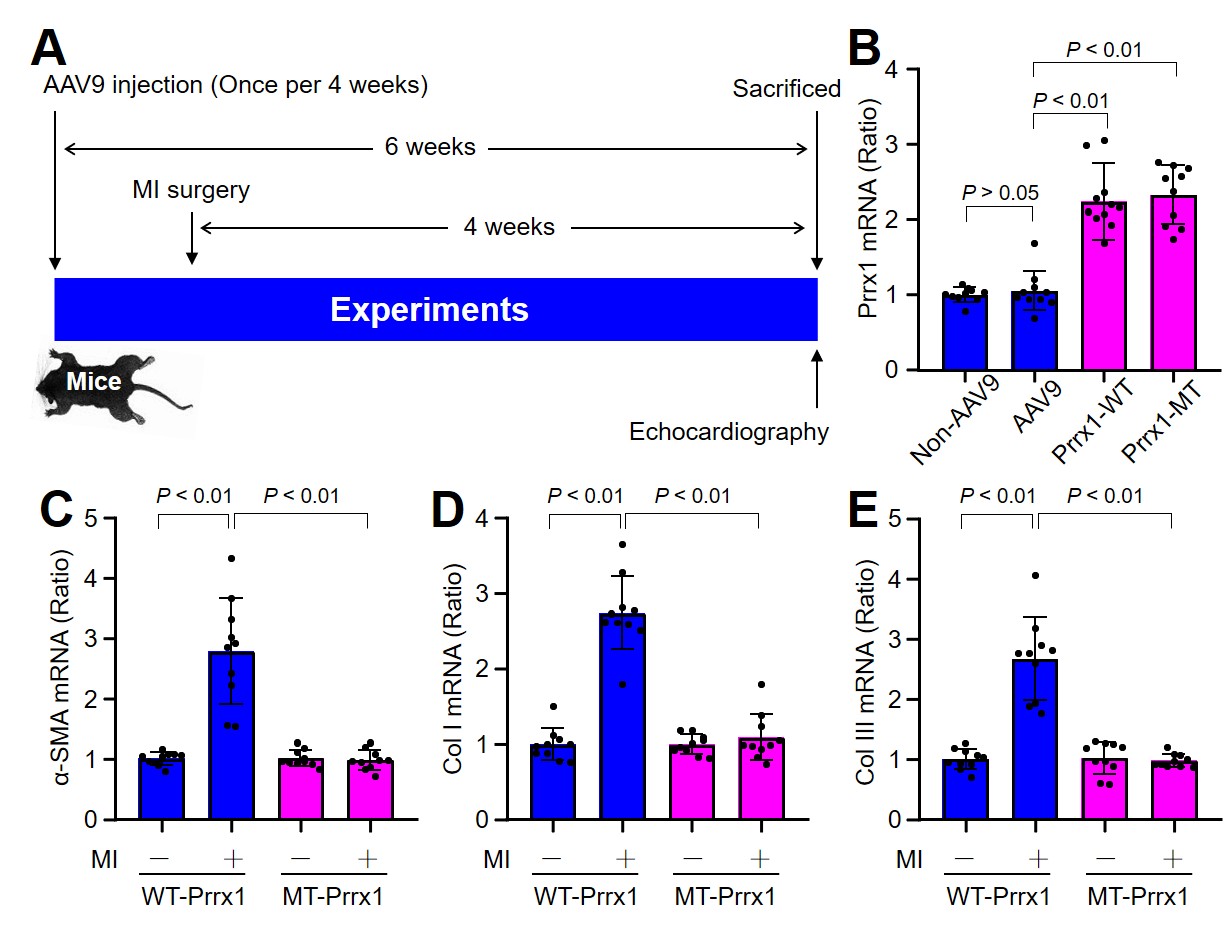


**Online Figure 6. Experimental protocols in AAV9-injected mice**. (**A**) mice were injected with AAV9 expressing Prrx1 (*WT*, C209R) per 6 weeks. Two weeks later, mice received the surgery of LADCA permanent ligation to induce myocardial infarction (MI). At the 28^th^ post operative day, echocardiography was performed to determine heart functions before scarified. At the end of experiment, heart tissues were harvested. (**B**) Cardiac fibroblasts isolated from mice were subjected to determine Prrx1 mRNA using quantitative PCR. N = 10 per group. A one-way ANOVA followed by Dunnett's test was used to determine *P* value. (**C-E**) Cardiac fibroblasts isolated from mice were subjected to determine gene expressions of α‑SMA in **C**, Col I in **D**, and Col III in **E** using quantitative PCR. N = 10 per group. A one-way ANOVA followed by Tukey’s HSD test was used to determine *P* value between two groups.


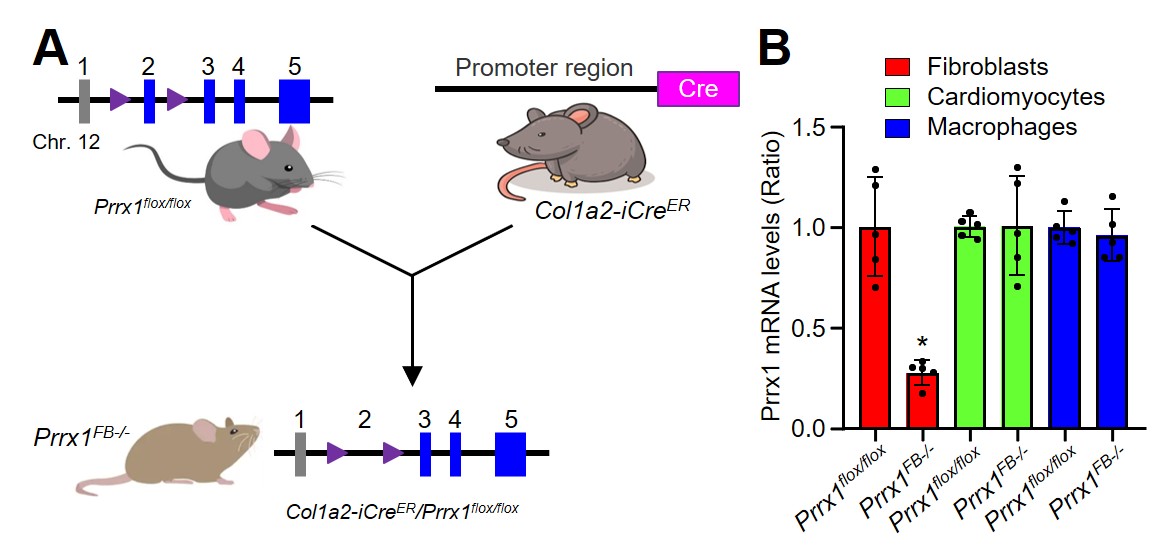


**Online Figure 7. Generation of fibroblast-specific Prrx1 gene knockout mouse**. (**A**) Schematic of fibroblast-specific Prrx1 gene knockout mouse. A clone containing mouse Prrx1 gene was modified by recombineering to insert loxP sites flanking exon 2 of Prrx1. *Prrx1^flox/flox^* mice were crossed with *Col1a2-iCre^ER^* mice to generate *Col1a2-iCre^ER^/Prrx1^flox/flox^* (*Prrx1^FB-/-^*) mice. (**B**) Primary fibroblasts, macrophages, and cardiomyocytes isolated from mice were subjected to determine Prrx1 mRNA using quantitative PCR. N = 10 per group. **P* < 0.05 *vs. Prrx1^flox/flo^* mice. An unpaired Student's *t* test was used to determine *P* value.


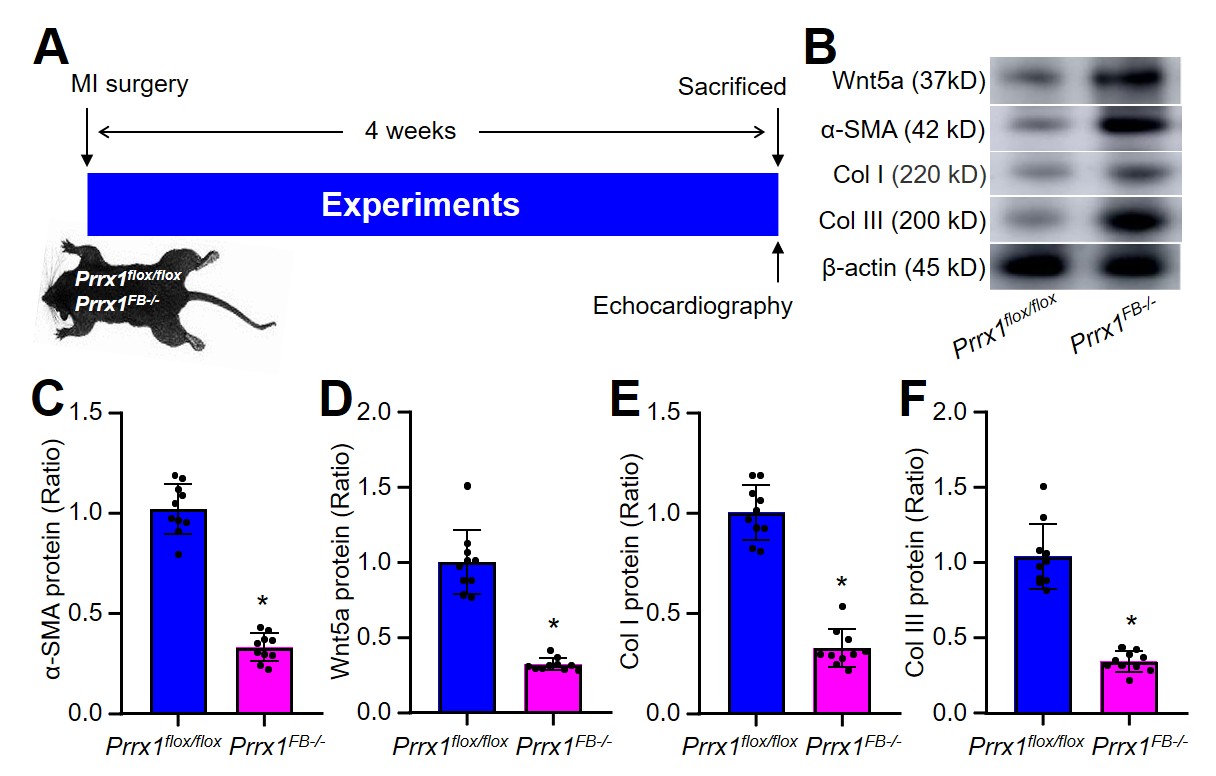


**Online Figure 8. Experimental protocols in fibroblast-specific Prrx1 gene knockout in mice.** (**A**) *Prrx1^flox/flox^* and *Prrx1^FB-/-^* mice received the surgery of LADCA permanent ligation. At the 28^th^ post operative day, echocardiography was performed to determine heart functions before scarified. At the end of experiment, heart tissues were harvested. (**B-F**) Cardiac fibroblasts isolated from mice were subjected to perform western blot analysis in **B**. Quantifications of Wnt5a in **C**, α‑SMA in **D**, Col I in **E**, and Col III in **F** were performed. N = 10 per group. An unpaired Student's *t* test was used to determine *P* value.


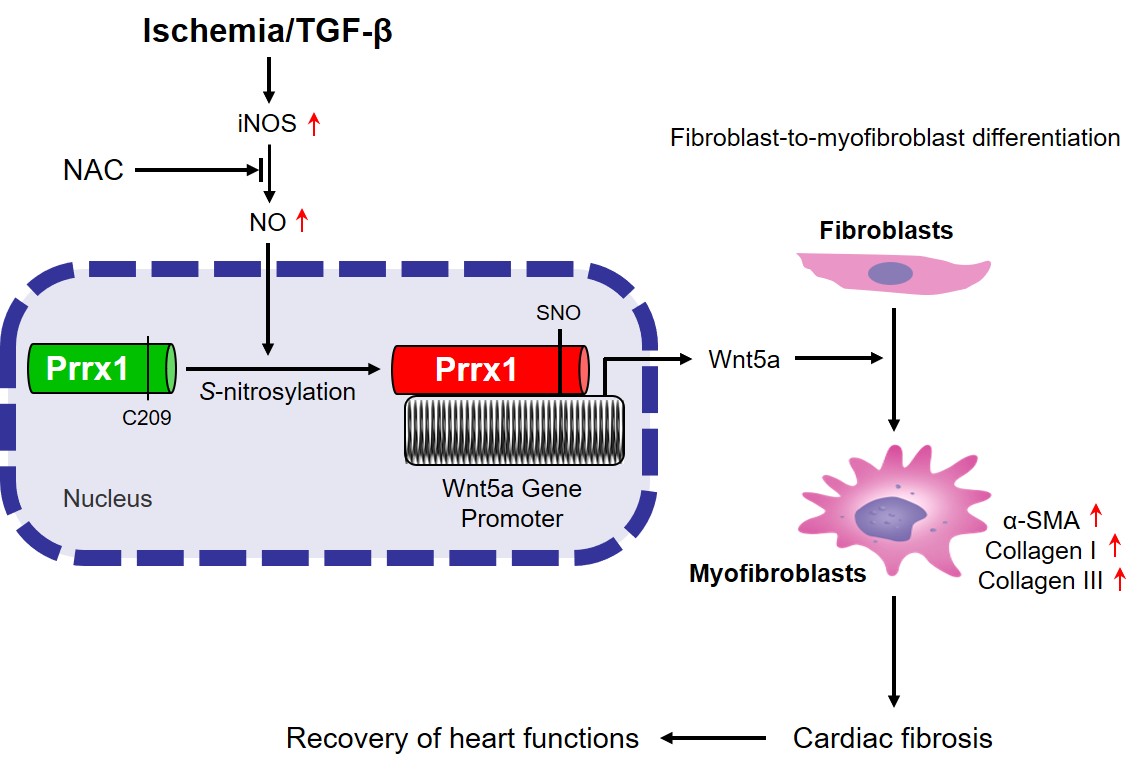


**Online Figure 9. Role of Prrx1 *S*-nitrosylation in cardiac remodeling following myocardial infarction (MI)**. In the post-ischemic heart, TGF-β activates Prrx1 transcriptional activity to upregulate Wnt5a gene transcription through Prrx1 *S*-nitrosylation at C207/C209. Activated Wnt5a signaling induces fibroblast-to-myofibroblast differentiation of cardiac fibroblasts, contributing to cardiac remodeling and the delayed recovery of heart function.

**Online Table 1. Demographic data for patients with one-year post-MI**

|  | **Non** | **MI** | ***P* value** |
| --- | --- | --- | --- |
| Patient numbers (N) | 15 | 15 | > 0.05 |
| Male (n) | 6 | 7 | > 0.05 |
| Age (years) | 67 ± 10 | 72 ± 13 | > 0.05 |
| Body mass index (kg/m^2^) | 23.1 ± 2.6 | 24.8 ± 3.9 | > 0.05 |
| Systolic blood pressure (mmHg) | 131 ± 23 | 139 ± 27 | > 0.05 |
| Diastolic blood pressure (mmHg) | 79 ± 16 | 71 ± 15 | > 0.05 |
| Heart rate (bpm) | 77 ± 19 | 86 ± 17 | > 0.05 |
| Fasting plasma glucose (mM) | 5.6 ± 1.7 | 5.3 ± 1.4 | > 0.05 |
| Homocysteine (μM) | 5.1 ± 1.2 | 6.4 ± 2.2 | > 0.05 |
| Triglyceride (mM) | 1.9 ± 0.3 | 2.1 ± 0.2 | > 0.05 |
| Total cholesterol (mM) | 4.2 ± 1.1 | 4.3 ± 1.0 | > 0.05 |
| High-density lipoprotein (mM) | 1.3 ± 0.2 | 1.0 ± 0.1 | > 0.05 |
| Low-density lipoprotein (mM) | 2.8 ± 0.6 | 3.4 ± 0.7 | > 0.05 |
| Smoking (n) | 10 | 2 | < 0.05 |
| Drinking (n) | 7 | 1 | < 0.05 |
| Hypertension (n) | 6 | 4 | > 0.05 |
| Stroke | 1 | 1 | > 0.05 |
| Serum NO (μM) | 36 ± 11 | 47 ± 16 | < 0.05 |
